# Lethal Fighting in Nematodes Is Dependent on Developmental Pathway: Male-Male Fighting in the Entomopathogenic Nematode *Steinernema longicaudum*

**DOI:** 10.1371/journal.pone.0089385

**Published:** 2014-02-24

**Authors:** Annemie N. R. L. Zenner, Kathryn M. O'Callaghan, Christine T. Griffin

**Affiliations:** Department of Biology, National University of Ireland Maynooth, Maynooth, County Kildare, Ireland; VIB & Katholieke Universiteit Leuven, Belgium

## Abstract

Aggressive encounters occur between competitors (particularly males) throughout the animal kingdom, and in some species can result in severe injury and death. Here we describe for the first time lethal interactions between male nematodes and provide evidence that the expression of this behaviour is developmentally controlled. Males of the entomopathogenic nematode *Steinernema longicaudum* coil around each other, resulting in injuries, paralysis and frequently death. The probability of death occurring between pairs of males was affected by the developmental pathway followed, being much greater among males that had passed through the infective juvenile (IJ, or dauer) stage than among males that had not. Post-IJ males are found only in newly colonised hosts, typically with few competing males present. Killing those few competitors may secure valuable resources (both females and a host cadaver for nourishment of offspring). Non-IJ males develop in subsequent generations within a host cadaver, where the presence of many closely related male competitors increases the risk:benefit ratio of fighting. Thus, passage through the IJ stage primes males for enhanced aggression in circumstances where this is more likely to result in increased reproductive success. Fighting occurred between males developing in mixed-sex social groups, indicating that it is an evolved trait and not an abnormal response to absence of females. This is supported by finding high mortality of males, but not of females, across a range of population densities in insect cadavers. We propose that these nematodes, with their relatively simple organization, may be a useful model for studies of aggression.

## Introduction

Fatal fighting is rare in the animal kingdom. In most cases, encounters between rivals for a mate or resources do not result in serious injury or death, but are settled by displays or trials of strength [1] [2]. The most dangerous fights are found in invertebrates. Males both of fig wasps and of the parasitoid *Melittobia* use their mandibles as weapons, severing limbs and decapitating rivals [3] [4] [5] [6], [7]. Examples of severe fighting resulting in death are also found amongst spiders [8] [9] and ants [10] [11], but have not previously been reported for any species of nematode. Theory predicts that fatal fights generally occur when individuals compete over a limiting resource (including females) that has a major impact on their lifetime reproductive success [2]. Factors favouring the evolution of fatal fighting include a small expected future value relative to the present, such as lower expectation of mating in the future relative to the present [2] and distribution of a resource in compact valuable masses, especially when the competitors cannot leave the resource. These conditions are experienced by fig wasps and *Melittobia*, for example [3] [2] [12], and such circumstances also prevail for the entomopathogenic nematodes S*teinernema* spp, which develop and reproduce inside a killed insect host. We have observed *Steinernema* males coil around each other, and such encounters have frequently resulted in injury and death of one of the protagonists (see supplemental movies), leading us to suspect that these animals fight over resources.

*Steinernema* spp. spend most of their life cycle feeding and reproducing in the cadavers of insects that have been killed and digested with the aid of their bacterial symbiont *Xenorhabdus* spp. [13] [14]. Like many parasitic nematodes, transmission from host to host is by means of a specialised infective juvenile (IJ) which actively seeks out and enters insects. The long-lived, non-feeding IJ is analogous to the dauer juvenile of *Caenorhabditis elegans* and other free-living nematodes; like the dauer (but unlike most parasitic nematodes) the *Steinernema* IJ forms facultatively in response to crowding; otherwise, juveniles develop to adult within the host. Thus, there are two distinct developmental pathways, the route taken depending on environmental conditions at a critical point in early development [15]. A large insect may support several generations of *Steinernema*; juveniles experiencing good conditions develop directly to adult, while those experiencing crowding become IJs, leave the spent host and colonise a new one before becoming adult. Assuming a relatively synchronous invasion of the new host insect, adults developing from IJs will reach sexual maturity at approximately the same time. Females have a short fertile period, as they quickly become incapacitated by juveniles hatching inside them (*endotokia matricida*) [16]. These conditions – an enclosed resource with declining availability of receptive females - should result in strong competition between males.

Amongst free-living nematodes there are rare descriptions of potentially damaging male-male interactions, and these have been interpreted as failure of males to discriminate between males and females [17] [18] [19]. Inflicting damage on rivals may be less important in free-living nematodes, which can move in search of fresh opportunities instead of competing. Parasites by their nature are more constrained in their movement, but are also less amenable to study. Indirect evidence of intraspecific competition may be obtained from incidence data: in certain oxyurid parasites inhabiting cockroach guts typically only a single pair (or at most one male and several females) of a species occurs per host; it is hypothesised that both males and females produce sex-specific chemicals effecting the elimination of conspecifics from the host [20] [21] [22]. We know of no reports of harmful interactions in parasitic nematodes involving direct physical contact. However, intra-sexual conflict has been reported in a parasitic worm from a different phylum, the acanthocephalan (spiny headed worm) *Moniliformis dubius,* where males place cement on their rivals' genitals [23]. The authors argued that, as cementing effectively removes rivals from the reproductive population, this behaviour probably evolved through sexual selection, with cementing of the female vagina (as a copulatory plug) as a pre-adaptation [23]. If the lethal interactions we observe between males of *Steinernema* are adaptive (a product of natural selection), a form of lethal combat evolved to eliminate rivals, then we might expect these behaviours to vary in response to internal or external conditions, as in other animals competing for resources.

Here we describe lethal interactions between male *Steinernema longicaudum*, and provide evidence that this fighting behaviour is shaped by natural selection rather than an unintended by-product of poor sex recognition, by investigating the effect of experience and social environment on its expression. In particular, we test the following hypotheses:

1. 1: Developmental pathway affects the probability of fighting. Males developing from IJs invade a new insect in the presence of relatively few, possibly unrelated individuals and have a good chance of monopolizing the available females, and so may benefit hugely from fighting. Males developing directly to adult without passing through the IJ stage are those of subsequent generations, which will typically experience many rivals, including full siblings and other close relatives [24], and so have less to gain from fighting [25] [26] [27]. Thus, there may be selection for plastic expression of life history or behavioural traits in adults destined to encounter these divergent conditions [28].
2. 2: Males are less likely to fight when the number of competitors is high. Environmental factors that are known to influence the occurrence and intensity of fights between animals include the value of the resource and the presence of competitors. As the number of competing males increases, individuals can benefit from fatal fighting only if their chances of winning are much greater than their chances of losing, thus the frequency of fatal fighting should decrease with increasing male numbers [29] [30] [31] [32]. This is tested *in vivo* (in insects) and *in vitro*. *In vitro*, we vary both the number of males in all-male groups and also in mixed sex groups. This further allows us to confirm that fighting occurs when females are present, and to test whether the number of females influences the probability of lethal outcomes.

Most experiments are carried out in the semi-natural conditions of a drop of insect blood (haemolymph); this provides a three-dimensional environment for the worms whilst permitting observation. The incidence of fighting was monitored during the start of the experiments, but as fights did not always occur immediately we use the outcome of fighting (paralysis or death) after 24 hours as the main measure that damaging interactions occurred. Since our first hypothesis was supported, other experiments were conducted only with IJ-derived males.

## Materials and Methods

Nematode culture and life cycle: *Steinernema longicaudum* CB2B cultures were routinely maintained using standard procedures by passage through late instar *Galleria mellonella* (wax moth) larvae [33] at 27°C. Infective juveniles were stored in tap water at 20°C.

*Steinernema* spp are mutualistically associated with entomopathogenic bacteria *Xenorhabdus* spp. (*Xenorhabdus ehlersii* in the case of *S. longicaudum*; [34]). The IJ carries cells of the symbiont in a specialised intestinal vesicle [14]. Once in the insect haemocoel (body cavity) the IJ expels cells of the symbiont from its gut. These proliferate, leading to rapid death of the host [35]; in *S. longicaudum* death may occur within 24 hours. The nematodes feed on a soup of bacteria and digested insect contents. Adult males and females reproduce by cross-fertilisation. Males have terminal spicules, hardened needle-like appendages which are inserted into the female's vulva to open it during sperm transfer. As conditions deteriorate, IJs are formed and leave the host; hundreds of thousands of IJs can be produced in a single wax moth larva [24]. In *S. longicaudum*, first IJs begin to emerge from infected wax moths after 8 days at 27°C.

Experimental infections: Wax moth larvae were exposed on filter paper to varying numbers of *S. longicaudum* infective juveniles (10 s to 100 s per insect) and then incubated at 27°C for 2–4 days (*N* = 72 insects, in 13 independent experiments). Dead insects were dissected in Ringer's saline and the number of live and dead, male and female adults of the first generation was recorded. At any one dissection time, representative cadavers from each IJ concentration were dissected, to avoid confounding effects of incubation time. *Steinernema* females initially lay eggs, but after some time eggs hatch inside and kill the females [16]. *S. longicaudum* females do not normally have internally hatched eggs within 4 days of infection at 27°C.

Hanging drop cultures: Adult nematodes used in experiments were reared in hanging drops of insect haemolymph, so that their social experience could be controlled. These cultures were initiated using IJs from *G. mellonella* culture. IJs were surface sterilised using hyamine and transferred to a hanging drop of *G. mellonella* haemolymph [33], usually one IJ per 25 µl drop. The symbiotic bacteria, released by the IJ as it recovered from its arrested state, grew in the medium, providing suitable nutrition for the nematodes and suppressing contaminating micro-organisms [36].

Paired fights: Unless otherwise stated, experiments were carried out using naïve worms that had developed from IJ stage in a hanging drop of haemolymph and had not had prior social experience before the fight was staged. Two adult males were removed from their hanging drops and placed, in pairs, back in the drop from which one member of the pair had been taken. Single males were removed from their drops and replaced in the same drops, to control for natural mortality. All adults used in an experiment were always of the same age.

Immediate observations were normally made on a proportion of the worms for up to 60 min, during which fighting attempts were recorded. Observations were also made at various times; we routinely report the outcome at 24 hours, as paralysis or death. A worm was deemed paralysed if a portion of the body (varying in extent and location) was not moving (see Supplemental movies); there was frequently a distinct kink between paralysed and mobile portions. Death of a nematode was confirmed by re-examination of the nematode on the following day. Observations were made using a Nikon Optiphot compound microscope.

### Effect of developmental pathway on mortality of paired males

To produce the nematodes used in the experiment, an adult male that had matured alone in a hanging drop was placed together with an adult female; eggs were laid into the medium, where they hatched. To produce adult males that had not passed through the IJ stage, juveniles that were amongst the first to hatch were transferred into a hanging drop of haemolymph that had previously been inoculated with bacteria-rich haemolymph. As more progeny were produced the parental drops became crowded and IJs developed, as expected under such conditions [15]. IJs (which are morphologically different from non-IJs) were transferred individually to drops of conditioned haemolymph, as described above for the non-IJs. This provided both categories of adult males: two males that had passed through the IJ stage were placed together (*N* = 83), and two males that had not passed through the IJ stage were also placed together (*N* = 96). Single male controls of each type were set up at the same time (*N* = 166 for IJ and 117 for non-IJ).

### Mortality of *S. longicaudum* in multimale groups

Nematodes were reared individually from IJ and then different numbers of naïve males (1, 2, 4, 8, 16 or 32) were placed together in a drop (*N* = 27, 23, 10, 4, 2 and 3, respectively).

### Mortality of *S. longicaudum* reared in social groups

Drops of haemolymph were inoculated with varying numbers (up to 15) of surface-sterilised IJs. These were incubated at 23°C instead of the usual 27°C, in order to slow the rate of development and provide opportunity to observe the nematodes before and after killing occurred. The number and status of male and female adults was assessed after 4 days. Of the 69 drops where males were found, there was 1 male in 24 drops, with 2, 3, 4, 5 and 6 males in 20, 18, 1, 4 and 2 drops, respectively.

### Statistical analysis

Statistical tests were carried out using Minitab16.0. Where multiple worms were present (in insects and in multi-worm groups in hanging drops), comparison of mortality between categories was done using *t*-tests (two-tailed) for two categories, and ANOVA for comparisons of more than two categories. Data were transformed (arcsine square root or log(x+1)) prior to analysis. For experiments involving just two males per hanging drop, data were totaled across all replications and repetitions in time. Incidence of a state, e.g. paralysis, death or production of progeny in different treatments was compared by cross tabulation using chi-square. Fisher's exact test was used where one or more expected value was less than 5.

## Results

### Male mortality is high and declines with increasing population size in insects

**Figure 1:**
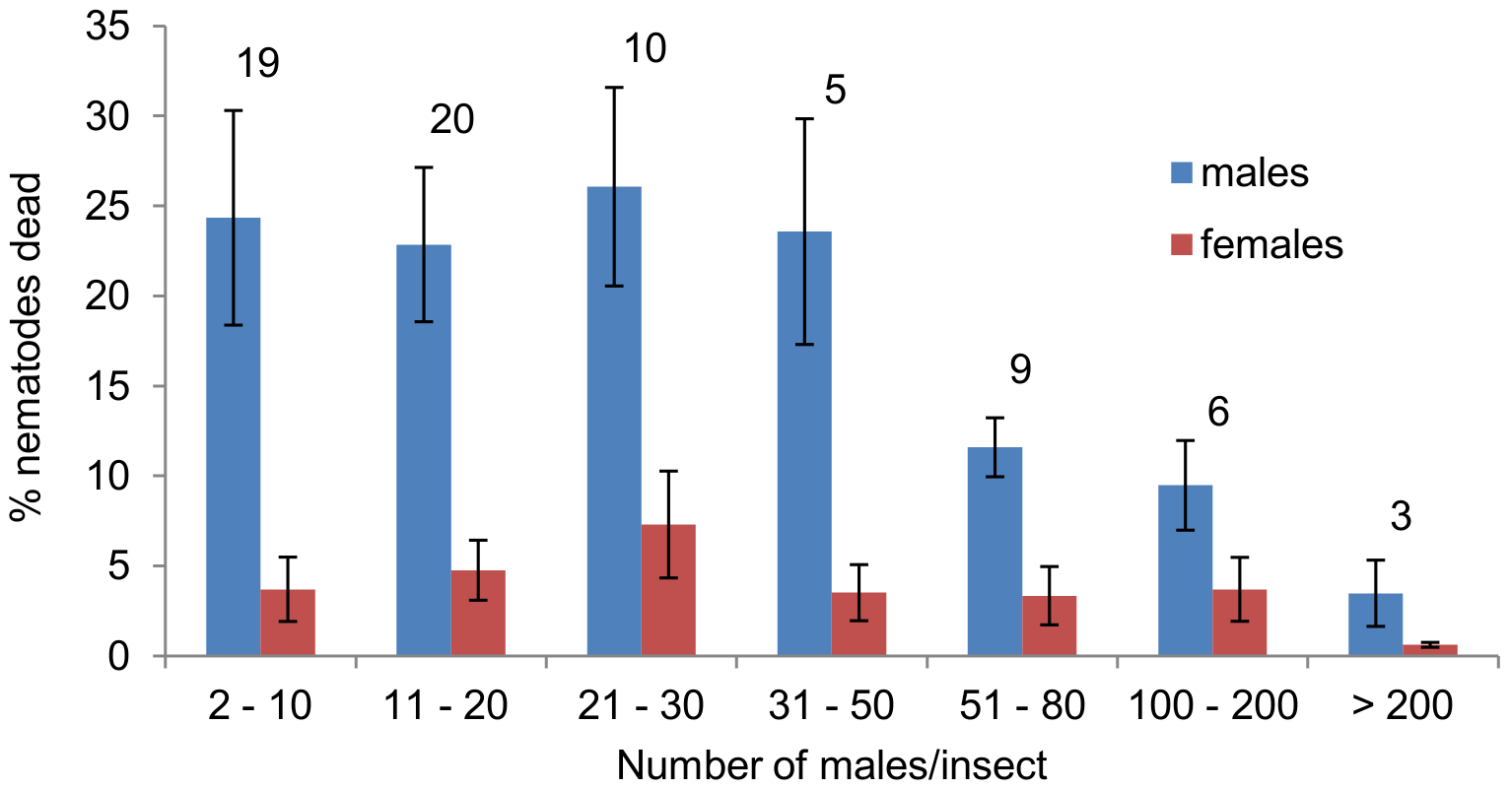
Effect of competitor density on killing in vivo. Percentage mortality (mean ± S.E.) of first generation adult males and females of the nematode *Steinernema longicaudum* in cadavers of the wax moth *Galleria mellonella*, with varying density, classed by number of males found in each cadaver. The number above the bar is the number of cadavers in each class.

Wax moth larvae were exposed to *S. longicaudum* IJs at a range of concentrations, in order to see the effect of worm density on killing in first generation males. The insects died within 2 days. When they were dissected 2–4 days after initial exposure to nematodes, the invading IJs had developed into adults, but a second generation of worms had not yet been produced. Overall, the proportion of males dead was four times higher than that of females (males: 370/3104; 11.9%; females, 110/3708, 3.0%), a highly significant difference between the sexes in mortality (chi-square  = 206.8, 1 d.f., *P*<0.001). Results were grouped by the number of males per cadaver (Fig. 1). Differences between all seven density classes were not significant (ANOVA F _6, 65_ = 1.14, *P* = 0.350). However, inspection of Fig. 1 indicates that male mortality averaged above 20% for each of the four categories with fewer than 50 males per cadaver (2–10, 11–20, 21–30 and 31–50 males/cadaver) but declined thereafter at each of the three higher densities (51–80, 100–200, and >200). The difference in male mortality in cadavers with fewer or more than 50 males was significant (*t* = 3.30, *P* = 0.002). Fewer than 6% females died in all categories (Fig. 1); there was no evidence that female mortality differed with male density (ANOVA F _6, 65_ = 0.57, *P* = 0.755;<50>*t* = −0.15, *P* = 0.884). Dissection of insects provides a snapshot in time, without opportunity to observe behaviours that might lead to death, and with limited opportunity to manipulate the social or other conditions that might influence mortality. For these reasons, subsequent experiments were carried out in drops of insect haemolymph.

### Fighting, paralysis and death between pairs of male *S. longicaudum* in vitro

**Figure 2:**
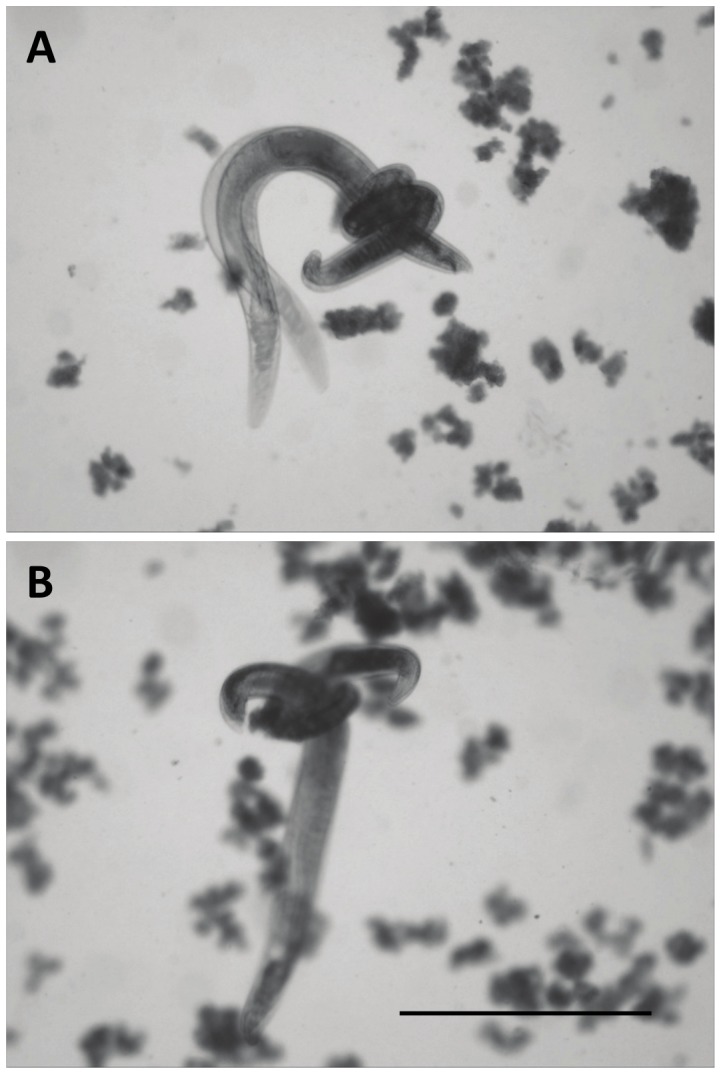
Fighting nematodes. Fighting in *Steinernema longicaudum* in a drop of insect haemolymph **A**. A male wrapped around the tail end of another male (the victim). The victim is moving rapidly at this stage, resulting in blurring of the image. **B**. The same pair ten minutes later. Here, the victim has slowed movement and is paralysed. Scale bar: 1 mm.

In order to explore the cause of death, nematodes were observed directly in hanging drops of haemolymph. When IJ-derived males were placed together in a drop, they frequently coiled around each other: typically, one male coiled its tail around the other male and pressed its copulatory spicules against it (Fig. 2A, B). Bouts of coiling lasted from seconds up to 20 min, and there could be repeated bouts. While the coiling is a behaviour resembling copulation (a male coils around the female and inserts its copulatory spicules into her vulva once it is located) and males also coil around inanimate objects of suitable size such as needles or surgical sutures, when a male coils around another male the victim is often partially or totally paralysed before being released (Fig. 2B; Supplementary Movies). Paralysed worms often maintained a “kinked” posture indicative of muscle contraction, in contrast to the straight or curved posture of dead worms. Partially paralysed worms were observed to twitch, or to engage in limited movements of a part of the body. Such worms sometimes recovered from the paralysis. When the victim was not gripped too near the tail, it could counterattack, pressing its own spicules to the cuticle of the aggressor coiled around it. Such counterattack could result in rapid release of the victim, and in some cases paralysis of the original aggressor (Movie S3). The cause of death is unclear, but injuries included ruptured cuticle and/or ruptured intestine and constriction of internal organs. However, some paralysed or killed worms bore no visible injuries.

**Figure 3:**
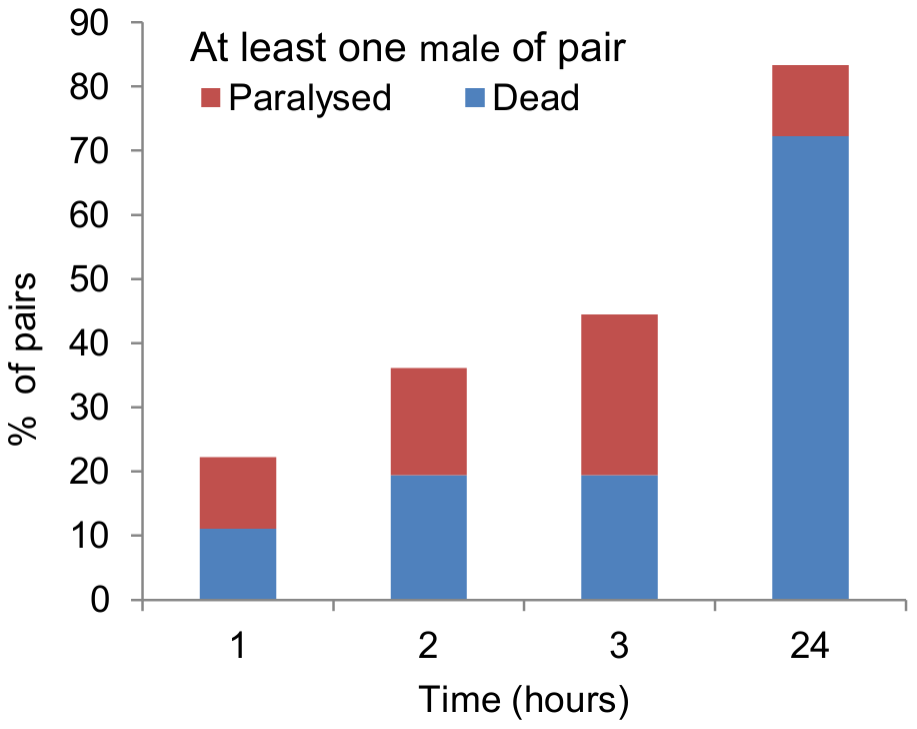
Time course of killing in IJ-derived males. Percentage of pairs of male *Steinernema longicaudum* in which one male was either paralysed or dead at various times after the males were placed together; N = 36 pairs. None of the 36 single male controls was paralysed or dead after 24 h.

Fights did not always occur immediately, but we did not detect any obvious pre-fighting behaviour or assessment. Death of one worm was the usual outcome when pairs of males were left together for 24 h. Figure 3 shows the time course of injuries in 36 pairs; by 24 hours, there was 1 male dead in 72% (26/36) of pairs, with one male paralysed but still alive in an additional 4 pairs (Figure 3). All of the 36 single male controls showed normal activity. The difference between pairs and singletons was highly significant both for death (chi-square  = 40.696, 1 d.f., *P*<0.001) and for paralysis and death (chi-square  = 51.429, 1 d.f., *P*<0.001). When virgin adult females were placed in pairs for 24 h, there was one female dead in 3% (2/75) of pairs, a value not different to that of single female controls (4%; 3/75; *P* = 0.817, Fisher's exact test), indicating that females do not kill each other and that death of males is not a simple consequence of crowding.

In order to relate death of a male in a pair to the observed “fighting” and consequent paralysis, additional pairs were observed until an encounter resulting in paralysis (either partial or total) of one of them was noted. The pair was then separated into “victor” and paralysed “victim” which were placed in separate drops. After 24 h, 70% (26/37) of victims and almost none (1/37) of the victors were dead (chi-square  = 36.44, 1 d.f., *P*<0.001), supporting the conclusion that where death of a male is observed after 24 h in a pair it is as a consequence of fighting between the males.

Since partially paralysed worms sometimes recovered, and it can be difficult to be certain that a nematode is dead, we assessed the ability of males that had no or reduced movement following a fight to inseminate a female. Victors and victims from 54 fights were separated and each was placed with two females: 98% (53/54) of victors and 15% (8/54) of victim males fertilised at least one female, as evidenced by production of progeny, indicating that most (though not all) of the worms scored as “paralysed or dead” were reproductively dead. The difference between victor and victim was highly significant (chi-square  = 76.282, 1 d.f., *P*<0.001).

### Developmental pathway affects subsequent mortality in pairs of males

**Figure 4:**
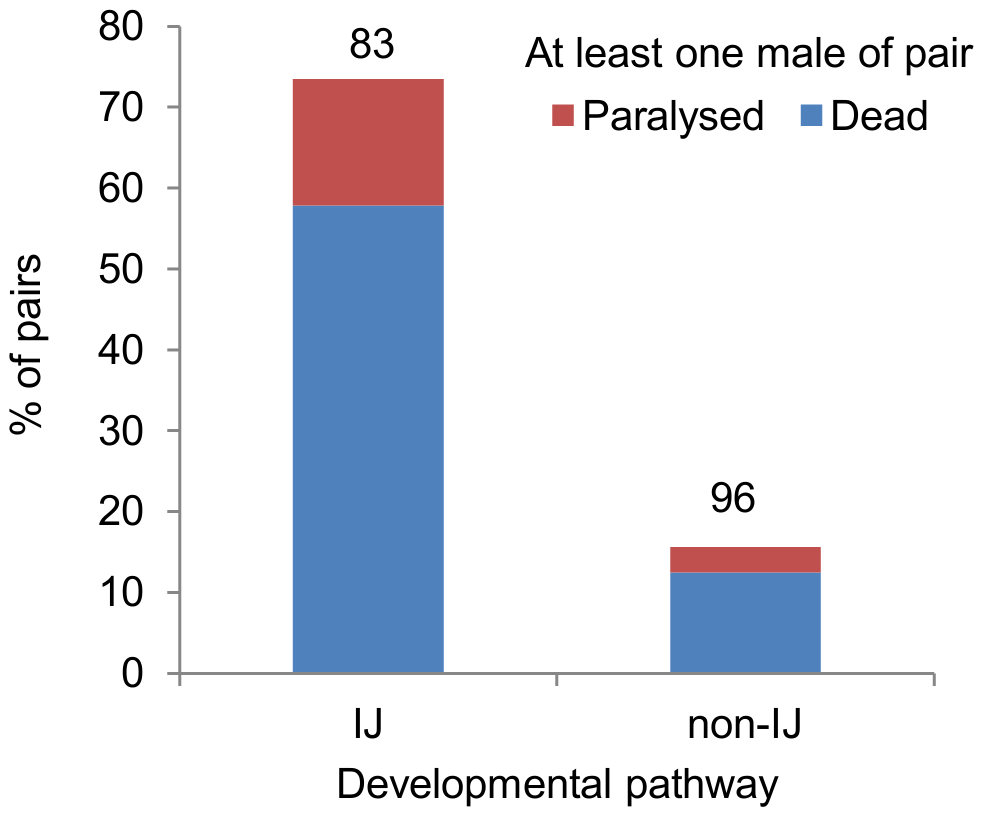
Developmental pathway affects killing. Effect of developmental pathway on fatal fighting in *Steinernema longicaudum*. Percentage of pairs in which at least one male was paralysed or dead 24 hours after they were placed together. Differences between the IJ and non-IJ pathways significant at *P*<0.001 (chi-square, 1 d.f. = 41.049 for dead, 61.016 for total affected (paralysed or dead). Numbers on the bars are numbers of pairs. A maximum of 3% of single males suffered paralysis or death, significantly different to pairs for IJ (chi-square  = 34.239, 1 d.f., *P*<0.001), but not for non-IJ (chi-square  = 2.064, 1 d.f., *P* = 0.151)

Fights were staged between pairs of adult males that had developed from IJs and between pairs which had followed the alternate developmental pathway, without passage through the IJ stage. When adult males derived from IJs were placed together in pairs for 24 h, there was at least one male dead in 58% of pairs, and evidence of severe fighting (at least one male paralysed or dead) in a total of 73% of pairs (Fig. 4); for non-IJ males, the corresponding values were 13% and 16% of pairs, a highly significant difference between developmental pathways for each parameter (death: chi-square  = 41.049, 1 d.f., *P*<0.001; paralysis and death: chi-square  = 61.016, 1 d.f., *P*<0.001; Fig. 4). No more than 3% of single male controls were dead for either developmental pathway (3/117 for IJ and 5/166 for non-IJ). There were no additional single males paralysed but not dead. The difference between pairs and singletons in the number of males dead was highly significant for males that passed through the IJ stage (chi-square  = 34.239, 1 d.f., *P*<0.001) and not significant for those that did not (chi-square  = 2.064, 1 d.f., *P* = 0.151).

### Males in single-sex groups suffer high mortality

Worms were reared individually from IJ and then different numbers of naïve males (up to 32) were placed together in a drop with no females. As all drops were approximately the same size, male number also reflects male density. The males coiled around each other within minutes. After 24 hours, mortality ranged from 50% in pairs to 78% in 8-male groups (Table 1). There was no difference in mortality due to number of males present (ANOVA, F _4, 37_ = 0.48, *P* = 0.747). In all groups of more than two males, mortality increased over the next 1–3 days, with a maximum of 94% dead in the 16 male groups (Table 1). Again, there was no difference in mortality due to number of males present (ANOVA, F _4, 37_ = 2.09, *P* = 0.103), though there was a trend for mortality to increase with increasing group size. The maximum percentage mortality expected due to fighting (assuming one male remains alive) is 50% in pairs and 96.9% in groups of 32 males. Two-four days after groups were formed, all but one of the drops with up to 16 males had only one male (or occasionally none) left alive (23/23, 9/10, 4/4 and 2/2 for drops with 2, 4, 8 and 16 males, respectively had 0–1 live male). The three drops with 32 males had 0, 3 and 6 males alive. However, eight of the nine surviving males appeared injured.

**Table 1:** Mortality (mean ± S.E.) of male *S. longicaudum* 24 h and 2–4 days after being placed in groups of 1–32.

		% males dead (mean ± S.E.)	
No. males/drop^1^	No. drops	24 hr	2–4 days
1	27	0	7.4
2	23	50.0±3.21	50.0±3.21
4	10	65.0±7.64	72.5±5.83
8	4	78.1±9.38	87.5±0.00
16	2	53.1±15.63	93.8±0.00
32	3	77.1±5.51	90.6±5.41

1 Males were reared separately in hanging drops each inoculated with one infective juvenile before being placed in groups.

### Males (but not females) reared in social groups suffer high mortality

The behaviour of nematodes developing on their own may differ from that of nematodes developing in social groups. In the previous experiments, mortality between pairs or groups of IJ-derived nematodes placed together after developing alone was higher than that seen in cadavers. Therefore an experiment was carried out to quantify male mortality when reared together from IJ under more normal social conditions, in mixed-sex groups (males and females, with up to six males per group). Mortality of males in groups with differing numbers ranged from 47.5% to 61.1% (Table 2); there was no effect of the number of males present (ANOVA, F _2, 42_ = 2.19, *P* = 0.125) on male mortality. The mortality of males overall was 54.7%, a value similar to that of males reared in isolation from IJ in the experiment on developmental pathway. Mortality of single males was 8% (2/24).

**Table 2:** Mortality (mean ± SE) of *S. longicaudum* males and females developing together for 4–5 days in hanging drops of insect haemolymph inoculated with infective juveniles.

		% nematodes dead (mean ± S.E.)	
No. males/drop	No. drops	Males	Females
1	24	8.3	5.9
2	20	47.5±5.71	6.3±5.09
3	18	61.1±4.04	5.6±0.00
4–6	7	58.6±9.31	4.8±2.65
All multi-male (2–6)	45	54.7±3.40	5.7±2.40

In addition to the males, there were up to 8 females per group. The mortality of females in multi-male groups (all groups with 2–6 males, combined) was nearly ten times lower than that of males in these groups (5.7% compared to 54.7%), a highly significant difference (*t* = 14.49, *P*<0.001). To ascertain if the number of females present affected the number of males that died, multi-male groups were classified as either “high combat” if only one (or no) male remained alive in a group, and as “low combat” if more than one male remained alive. The sex ratio (females: males) did not differ between levels of combat (high: mean ± SE 0.63±0.70, *N* = 34; low: 0.59±0.17, *N* = 11; *t* = 0.20, *P* = 0.842). There was also no difference between combat categories in the proportion of groups with at least one female present (low combat 63.6% (7/11), high combat 85.3% (29/34); Fisher's exact test, *P* = 0.19), indicating that the level of combat was not affected by the presence or absence of females in the group.

## Discussion

Mortality for male *S. longicaudum* was four times as high as for females in recently invaded insects. While we cannot directly observe what takes place inside an insect, males of this species can be seen to kill each other in the semi-natural environment of a drop of insect haemolymph. We infer that killing is the cause of high male mortality in the insect also, and argue that this behaviour has evolved to remove competitors.

Factors favouring the evolution of fatal fighting include competition for valuable resources, particularly compact, defensible resources which the males cannot leave [2], [3]. These conditions exist for *Steinernema* males. A single insect cadaver can support several generations and produce up to 300,000 nematodes, representing a substantial reward for a founding male that can monopolise all the founding females by dispatching the limited number of other males likely to be present in the founder generation resulting from invasion of the host by IJs. The high number of males in subsequent generations produced through the non-IJ pathway [24] means that reward to risk ratio for fighting would be much lower, as a higher number of opponents must be defeated. As predicted, there was a large difference in killing between males that developed from IJs and those that did not. In more than half of the pairs of IJ-derived males, one killed the other, while in pairs of males that had developed directly there was little evidence of killing. Since in our experiments males of both types were reared and tested under identical conditions, the difference in fighting must be due to developmental pathway (or to the conditions in early juvenile life that influenced the pathway) rather than to the prevailing conditions at the time of fighting. Thus, passage through the IJ stage primes males for enhanced aggression in circumstances where this is more likely to contribute to enhanced reproductive success. We see the difference in fighting between IJ-derived and non-IJ males as strong evidence that fatal fighting is an adaptive behaviour in these nematodes, evolved under selection pressure for reproductive success, and that male death is not an unintended outcome of same-sex sexual behaviour due to poor sex recognition [37].

Other evidence that mistaken identity is not the cause of the damaging male-male interactions in *S. longicaudum* comes from the extreme outcome of the interactions, the fact that they occur even in the presence of females and differ in form from copulation behaviour. The interactions also conform to several predictions based on theoretical assumptions regarding the adaptiveness of intraspecific fighting (Zenner & Griffin, unpublished). Lack of sex discrimination may be favoured where costs of a homosexual encounter are small [38], as appears to be the case in *C. elegans*. In some strains of *C. elegans*, males in all-male groups place a copulatory plug on the excretory pore of another male, resulting in shortened life-span, such that pairs of males had a median lifespan of 15 days compared to 20 days in males kept on their own [17]. In another free-living nematode, *Oncholaimus oxyuris*, where males inseminate females by puncturing the cuticle, males also insert their copulatory spicules into the anus or through the cuticle of another male [19]. Adverse effects of puncturing on male reproductive potential or lifespan were not investigated [19]. Clearly, male-male encounters are costly in *S. longicaudum*, resulting in immediate paralysis (Supplemental Movies) followed by death within hours; even the male originating the encounter may be killed. Sex recognition factors would be expected to evolve under this kind of selection pressure [39]. Animals may engage in sexual interactions with members of the same sex when members of the opposite sex are absent or scarce- the “prisoner effect”, but such behaviour is expected to disappear when members of the opposite sex are available [40]. In *C. elegans*, males plugged each other only in all-male groups [17], while in *S. longicaudum* attacks occurred under all social conditions: in pairs, in all male groups, and in mixed sex groups. Moreover, the nature and outcome of the encounter between two males differs from that between a male and a female. On encountering a female of its own species, a steinernematid male wraps its entire body around it and frequently changes position, seeking the vulva. In contrast, on encountering another male he wraps only his tail end around the victim's body, does not shift position, but grips tightly, until the victim reduces movement. We found no evidence of negative interactions between females, either by direct observations or from the outcome of encounters. Amongst animals in general, it is males that compete as they can increase their reproductive output by removing rivals [1] [2]; moreover, in *Steinernema,* the male's ability to coil tightly around another worm and possession of hardened spicules, both features evolved for copulation, provide it with “weaponry” lacking in females.

The reduced male mortality at high density of competitors (cadavers occupied by more than 50 males) is as predicted; at such high density of competitors there is lower probability that a victorious male will increase its probability of mating, and fighting wastes time that could be spent in mating [29] [41] [42]. However, there was little evidence that increased competitor number reduced fighting in haemolymph, either when males were reared in mixed-sex groups or placed together in multi-male groups. It is difficult to make direct comparisons of crowding between drops (about 25 µl liquid) and insects weighing on average 250 mg. One possible reason for the difference between in vivo and in vitro experiments is that the restricted area of the drop, and its rather uniform liquid medium, provides less opportunity for a male to avoid an attack than does the insect cadaver. As male density increases so too does the encounter rate, counteracting reduced aggression at intermediate densities [12] [29] [41]. This is supported by the fact that the overall kill rate was higher in vitro than in vivo.

The level of fatal fighting recorded in our experiments may be unusually high as a result of the unnatural experimental procedures. Males used in most experiments were reared in isolation, which may affect adult behaviour. Rearing *C. elegans* in isolation had widespread cellular and behavioural consequences, and affected the development of neuronal connectivity [43], while *Drosophila* that had experienced social isolation displayed increased aggression [44]. However, while social isolation during development may have exacerbated aggression, males reared in mixed-sex groups also killed a high proportion of competitors. A second factor that may have exacerbated the level of fatal fighting overall is the relatively small size of the arena, with less opportunity for retreat [9] [45] [46] than may be found in many host insects. Nevertheless, there is evidence that killing takes place in a wax moth larva- a relatively large insect in which it should be possible for worms to avoid each other.

Fatal fighting in *S. longicaudum* is profoundly influenced by the male's developmental experience and to a lesser extent by its environment including population density (in insect), as shown here, as well as by its mating status, prior victory and residency (Zenner & Griffin, unpublished data). There are thus several factors influencing the male's decision whether to fight. Most studies on aggression and fighting have been done with animals with more complex nervous systems, such as vertebrates and insects. However, even animals without a centralised nervous system such as anemones appear to use logical decision rules in contests [47] [48]. Despite the relatively small number of neurons compared to other animals, the *C. elegans* male is capable of surprisingly complex and sophisticated sexual behaviour that involves integrating information from the environment and the worm's physiological state [49] [50] [51]. Thus, it is not surprising that a “simple” parasitic nematode is capable of such sophisticated behavioural strategies as are described here.

Across taxa, early experience profoundly affects the behavioural phenotype expressed by animals in later life [52] [53]. The probability of *Steinernema* males fighting was greatly influenced by the developmental pathway they had followed - whether or not they had passed through the IJ stage, which is triggered at an early stage in development. In *C. elegans*, passage through the functionally analogous dauer stage resulted in adults with differences in life history traits, including extended adult life span and increased progeny production compared to non-dauer adults [54]; an example of developmental history programming adult physiology and behaviour through epigenetic mechanisms [54] [55]. The availability of a simple tractable nematode model may facilitate the study of effects of developmental and social history on aggression.

There is a growing body of evidence that similar neural mechanisms are at work from worms to humans even in coordinating complex behaviours [56], making the relatively simple nematodes attractive for studies of fundamental processes underpinning for example fear and anxiety, and diseases such as Alzheimer's and Parkinson's [57] [58]. The extensive body of knowledge of *C.elegans* genetics, neurochemistry and neural circuitry has allowed tremendous advances to be made regarding the neural underpinning of behaviour from environmental stimulus to behavioural expression, influenced by motivational state [59] Increasingly, studies on other nematodes complement and build on the knowledge-base of *C. elegans*. *Steinernema* spp are increasingly used as model organisms for addressing questions regarding symbiosis and parasitism, for example [60] [61]. Genomes of five *Steinernema* species have been sequenced and annotated [61] facilitating this investigation. We have detected fighting in several *Steinernema* species in addition to *S. longicaudum* (O'Callaghan, Zenner, Griffin, unpublished data), suggesting that it is a characteristic of the genus. Our demonstration of aggressive interactions in *Steinernema* means that a nematode is now available to complement arthropod models [62] [63] for testing hypotheses about aggression, and exploring neural mechanisms and proximal cues in fighting.

## Supporting Information

Movie S1 Tailgrapple: This is a typical fight between two males. Initially, both are moving normally. Then one wraps its tail end around the tail end of the other male. The “wrap” lasts only a few seconds. The released victim gradually ceases to move the posterior part of its body, so that by the end of the clip only the anterior one-third is moving normally. The victor contacts the victim several times using its head or tail, as if checking for movement.
(AVI)

Movie S2 Headgrab. This movie opens with two males moving normally. After about a minute, one male wraps its tail around the anterior end of the other male and coils tightly. Initially, the victim thrashes strongly as if to gain release, but within 30 seconds has almost ceased moving. The victor unwinds partly and briefly touches the victim's tail with its head, as if to check that it is incapacitated. It then fully releases its hold. The victor contacts the victim again several times. The victim remains largely inactive and abnormally bent at both head and tail ends.
(AVI)

Movie S3 Successful counterattack. When this movie opens, one worm has its tail coiled about the tail of the other; soon, it appears that the two males are entangled by their tails. For a short while, we can see that the worm on right of frame is grasped by the worm on left, but at around 40 seconds into the clip this changes, so that worm on the left is now the victim. When released, the victim is moving feebly; the victor leaves.
(AVI)

Movie S4 Unsuccessful counter-attack. This movie opens with two males moving normally. One wraps its tail around the anterior end of the other, leaving the victim's tail free. The victim now brings its own tail into contact with the aggressor's body and is soon released. However, it appears to be too late, as within seconds of being released the victim has ceased activity apart from some weak movements of the head.
(AVI)
